# Multidimensional Urbanization Effects on Spontaneous Plant Diversity in a Cold Climate Megacity

**DOI:** 10.3390/plants14172753

**Published:** 2025-09-03

**Authors:** Xingyuan Wang, Congcong Zhao, Mingyu Yu, Yuandong Hu, Zhiwen Gao

**Affiliations:** 1 College of Landscape Architecture, Northeast Forestry University, Harbin 150040, China; wangxingyuan0302@163.com (X.W.); zhao_congcong@nefu.edu.cn (C.Z.); 2 College of Mechanical and Electrical Engineering, Northeast Forestry University, Harbin 150040, China; 15504391961@163.com; 3 XAUAT-UWA International Joint Lab on Urban Biodiversity and Design, Xi’an University of Architecture and Technology, Xi’an 710055, China; 4 Faculty of Geography, Yunnan Normal University, Kunming 650500, China

**Keywords:** spontaneous plants, multidimensional urbanization, habitat heterogeneity, building volume, urban green space

## Abstract

Urbanization profoundly transforms ecosystems, often resulting in habitat loss and biodiversity decline. Urban spontaneous plants, which are established naturally without human intervention, play a critical role in urban ecosystems by providing habitats, mitigating urban heat islands, and acting as indicators of environmental changes. Multidimensional urbanization, encompassing vertical and horizontal scale, exerts a significant influence on the biodiversity of green space. While previous studies have extensively examined the effects of horizontal spatial scales (such as land use and population density), the impacts of vertical spatial scales remain understudied. To elucidate the spatial patterns and driving factors of spontaneous plant diversity under multidimensional urbanization, we conducted a comprehensive survey of spontaneous plants across Changchun, a rapidly urbanizing city in northeast China. We established 1147 herbaceous plots within 245 urban green space patches across 38 sites and analyzed the effects of multidimensional urbanization metrics on spontaneous plant diversity. A total of 408 species of spontaneous plants were recorded, with herbs as the dominant life-form (89.2%), 322 are native species (78.9%), and 21.1% non-native species (of which 65.1% are invasive), primarily dispersed by autochory. Significant differences in plant diversity indices were observed across various urban green spaces and habitat types in Changchun, with native plant diversity generally highest in square green spaces and scrub gaps, while non-native plant diversity was most prominent in brownfield sites and showed no significant variation among habitat types. Regression analyses revealed that, in addition to patch characteristic factors (including patch area, perimeter–area ratio, and landscape shape index), the richness of total, native, and autochorous spontaneous plants was primarily influenced by vertical urbanization (as indicated by building volume), with building volume positively associated with species richness. In contrast, the richness of non-native and anemochorous plants was also significantly affected by horizontal urbanization factors, such as the proportion of impervious surface within a 100 m buffer zone and distance from patch to city center. The results reveal distinct spatial patterns of spontaneous plant diversity driven by both urbanization of horizontal spatial scales and vertical spatial scales. Our study provides new insights into the interplay between multidimensional urbanization and biodiversity, offering a theoretical foundation for integrating biodiversity conservation into sustainable urban planning and ecosystem management.

## 1. Introduction

As global urbanization intensifies, the rapid expansion of urban areas has led to a substantial shift in land use patterns, with ongoing loss and fragmentation of natural habitats and a decline in biodiversity [1,2]. Urban development often transforms diverse ecosystems into impervious surfaces, reducing habitat availability and disrupting ecological processes [3]. Relevant studies have shown that the rapid urbanization of Changchun has led to a decline in the landscape ecological security of peri-urban cultivated land [4], with the habitat quality gradually deteriorating [5]. These changes significantly impact biodiversity, as urban areas increasingly support both human populations and species that have adapted to urban environments [6]. However, urbanization also offers opportunities to mitigate biodiversity loss through the strategic planning and management of green spaces [7]. Understanding the complex interplay between urbanization and biodiversity is crucial for developing strategies to conserve biodiversity and maintain ecological functions in urban ecosystems.

Urban spontaneous plants, defined as plant species that establish naturally and are not artificially planted in urban environments, are an indispensable part of the urban ecosystem [8,9,10,11]. These resilient organisms colonize almost all habitats in urban areas, from abandoned lands to parks, from road gaps to rooftops, demonstrating remarkable adaptability and stress tolerance under variable conditions [12,13,14,15]. Spontaneous plants also serve as pioneer species for ecological restoration and as sensitive indicators of environmental changes [11]. Their sensitivity to environmental changes makes them effective indicators of urban environmental quality, such as pollution and habitat fragmentation [16]. Additionally, research highlights their ecological importance as they provide critical ecosystem services, including improving soil structure, mitigating urban heat island effects, and supporting wildlife by offering food and habitat [17]. Researchers have explored the multifunctionality of spontaneous plants, emphasizing their contributions to landscape and natural succession in urban areas [18]. Furthermore, their aesthetic value in creating visually appealing, naturalistic urban spaces has been increasingly acknowledged [19].

The effects of urbanization on biodiversity have been extensively studied, particularly focusing on horizontal dimensional urbanization, such as land use change [20], population density [21], and impervious surface coverage [22,23]. For example, research has shown that increasing urban sprawl leads to habitat loss and fragmentation, driving declines in species richness and shifts in plant species composition [10]. Furthermore, horizontal dimensional urbanization metrics, such as urban area size and land-use intensity, have been widely used to explore patterns of species richness, functional traits, and ecosystem services in urban environments [24]. These horizontal dimensional urbanizations have provided valuable insights into patterns of biodiversity loss and adaptation in urban environments. Vertical dimensional urbanization features are also increasingly prominent in rapidly urbanizing regions, where vertical and uneven expansion have become defining characteristics of urban development [25]. The vertical dimension of cities—including building height, urban volume, and vertical green infrastructure—not only creates novel habitats and alters microclimates but also influences the dispersal and colonization patterns of spontaneous plants. For instance, recent studies have examined the effects of building volume and height variation on urban plant diversity and have incorporated three-dimensional building metrics, such as building volume and its coefficient of variation, into models assessing spontaneous species richness [26]. Other research has explored how vertical green infrastructure and building height can influence microclimates and plant colonization patterns [27]. Incorporating three-dimensional urban features, such as building volume and its spatial variation, into analytical frameworks is therefore crucial for understanding the complexity of urban ecosystems and for informing urban planning strategies that reconcile vertical growth with biodiversity conservation.

As one of the central cities in Northeast China, Changchun has a green coverage rate of 42.2% in its built-up area. The city’s urban green spaces are characterized by relatively high species richness, including a mix of native and introduced plant species, but their spatial distribution remains uneven [28,29]. However, rapid urbanization over the past 40 years has led to dramatic changes in both the horizontal and vertical dimensions of the city. Horizontally, the area of construction land in Changchun has expanded significantly, with urban built-up areas increasing from approximately 89 km^2^ in the 1980s to over 676 km^2^ by 2023 [30]. This expansion has resulted in the conversion of large amounts of agricultural and natural land into urban infrastructure, contributing to habitat fragmentation and the loss of green space connectivity [31].

Vertically, Changchun has experienced a marked increase in both the number and density of high-rise buildings, especially in the central and newly developed districts. The average building height in the city center was less than 10 m in the early 1980s, while nowadays many residential and commercial complexes have more than 20 floors. This vertical growth has reshaped the urban skyline and led to the emergence of new microclimates, such as urban heat islands and wind corridors, which can influence the distribution and colonization patterns of urban plant species [32]. These vertical-dimensional urban features not only create novel habitats for spontaneous vegetation but also present new challenges and opportunities for biodiversity conservation in highly urbanized environments [33].

Despite these transformations, the effects of different urbanization dimensions on the urban biodiversity of green space remain poorly understood. To address this knowledge gap, we formulated the following research hypotheses: (1) Rapid urbanization, characterized by increased impervious surface, will lead to a reduction in spontaneous plant diversity within urban green spaces; (2) vertical dimensional urbanization features, such as building volume and coefficient of variation of building volume, will have distinct effects on the spatial distribution and composition of spontaneous plants compared to two-dimensional urban expansion. To test these hypotheses, we conducted a comprehensive survey of spontaneous plants across the main urban area of Changchun and established 245 urban green space patches and incorporated both horizontal and vertical dimensional urbanization metrics to analyze their impacts on spontaneous plant diversity. Our study aims to uncover the spatial patterns and driving factors of spontaneous plant diversity under the influence of multidimensional urbanization. By providing a detailed understanding of these dynamics, this research offers a robust theoretical foundation for integrating biodiversity conservation into urban planning and management, contributing to the sustainable development of Changchun’s urban ecosystems.

## 2. Results

### 2.1. Species Composition

This study investigated a total of 408 spontaneous plant species in Changchun, distributed across 244 genera and 65 families. Herbaceous plants clearly dominated the spectrum of spontaneous plant life-forms in Changchun. Specifically, perennial herbs comprised 186 species (45.6%), while annual and biennial herbs accounted for 178 species (43.9%). Ferns were represented by a single species, *Equisetum arvense* (Supplementary Materials).

**Figure 1:**
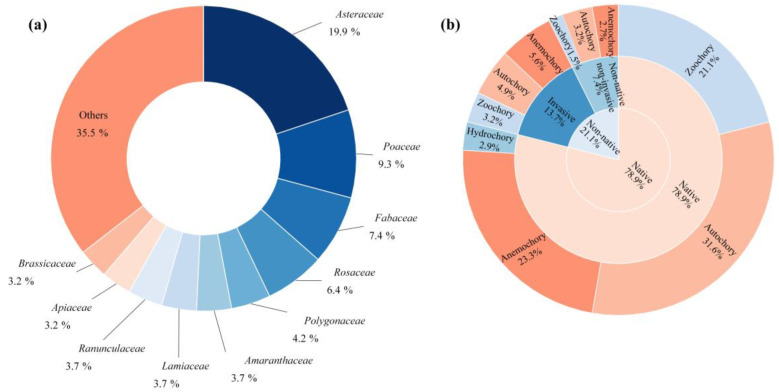
(**a**) Composition of families of spontaneous plants in Changchun. (**b**) The composition of species origins and dispersal modes of spontaneous plants in Changchun.

Among these families, eight contain more than 15 species (Figure 1), accounting for 125 genera (51.2% of all genera) and 237 species (58.1% of all species). The family *Asteraceae* had the highest number of species (81), followed by *Poaceae* (38) and *Fabaceae* (30). The species with the highest occurrence frequency is *Taraxacum mongolicum* (shown in 94.3% patches).

Of the total species, 322 (78.9%) were native plants, while 86 (21.1%) were non-native. Among the non-native species, 56 were identified as invasive, comprising 65.1% of the non-native plants. Among invasive plants, *Erigeron canadensis* (shown in 77.6% patches), *Ambrosia artemisiifolia* (47.8% patches), and *Amaranthus retroflexus* (27.3% patches) showed high frequency. Notably, two invasive species were among the top 10 most frequently occurring species in this survey: *Erigeron canadensis* (found in 77.6% of patches) and *Galinsoga quadriradiata* (79.6%, Figure 1).

Regarding dispersal modes, autochory was the most common, with 162 spontaneous species relying on this mechanism, including 20 invasive species (12.3% of autochorous plants). In contrast, hydrochory was the least common dispersal mode among the surveyed species. Among invasive plants, anemochory is the main dispersal mode (Figure 1b).

### 2.2. Diversity Pattern of Spontaneous Plant Diversity in Changchun

**Figure 2:**
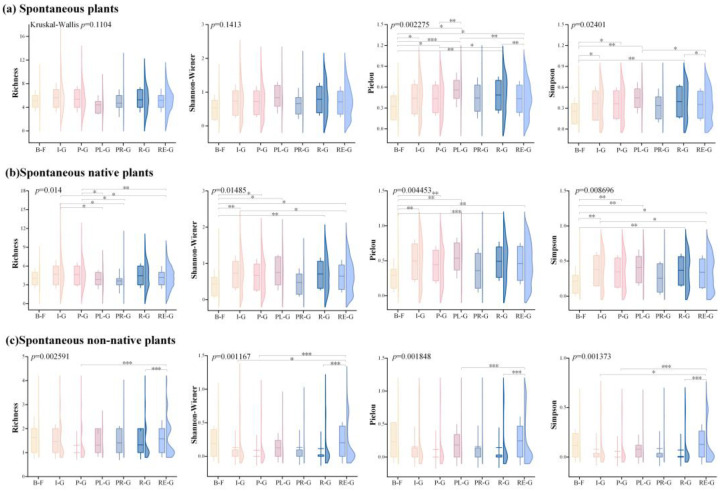
Diversity indices of various spontaneous plant categories in various kinds of green spaces. Note: *** *p* < 0.001; ** *p* < 0.01; * *p* < 0.05. B-F: brownfield site; I-G: affiliated green space; P-G: park green space; PL-G: square green space; PR-G: protective green space; R-G: road green space; RE-G: residential green space.

In the seven categories of urban green spaces, the diversity of total spontaneous plants, native plants, and non-native plants shows that the Shannon-Wiener diversity index, the Pielou evenness index, and the Simpson dominance index of total spontaneous plants and native plants had the highest mean values in the square green space. The Pielou evenness index and the Simpson dominance index for total spontaneous plants were significantly different (*p* < 0.05) across green space types, as were all four diversity indices for spontaneous native plants. The mean values of the Shannon-Wiener diversity index, the Pielou evenness index, and the Simpson dominance index were highest in the residential green space, whereas the Richness index for spontaneous non-native plants had the highest mean value in the brownfield site. Among the various types of green spaces, there was a significant difference (*p* < 0.05) in all four diversity indices for spontaneous non-native plants (Figure 2).

**Figure 3:**
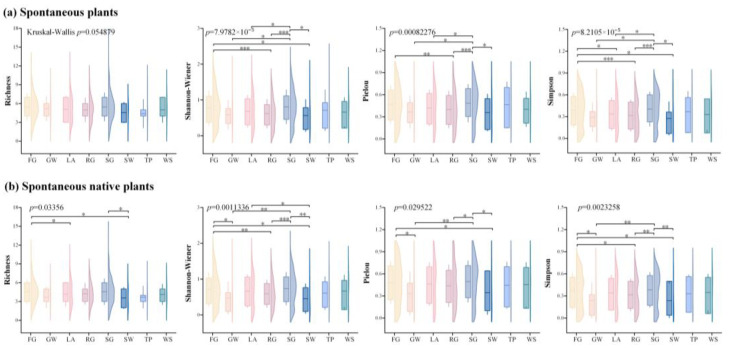
Diversity indices of several spontaneous plant species on various types of habitats. Note: *** *p* < 0.001; ** *p* < 0.01; * *p* < 0.05. FG: forest gap; GW: gravel wasteland; LA: lawn; RG: road gap; SG: scrub gap; SW: soil wasteland; TP: tree pool; WS: water side.

The diversity of spontaneous plants in the eight different urban habitat types revealed that the mean values of the Shannon-Wiener diversity index and Richness index of spontaneous plants were the highest in forest gap; Pielou evenness index (0.48) and Simpson dominance index (0.40) of spontaneous plants were highest in scrub gap. Additionally, there were significant differences (*p* < 0.05) in the mean values of these three indicators between the various urban habitat types. The scrub gap had the greatest mean values of the Shannon-Wiener diversity index, Pielou evenness index, and Simpson dominance index of spontaneous native plants. Notably, there was a significant difference (*p* < 0.05) in all four diversity indices between urban habitats. For non-native plants, there were no discernible differences between the various urban habitat types in the four diversity indices (Figure 3).

### 2.3. Environmental Drivers of Spontaneous Plant Diversity

Linear regression modeling indicated that patch characteristics were key factors influencing the richness of all plant groups. Specifically, the patch perimeter–area ratio (PA) was significantly negatively correlated with the richness of all groups (*p* < 0.05), while the landscape shape index (LSI) showed a significant positive correlation with the richness of all groups. Patch area was positively correlated with zoochorous species richness. Woody layer cover (TC) and disturbance intensity (D) were both significantly negatively associated with the richness of non-native plants (*p* < 0.05).

**Figure 4:**
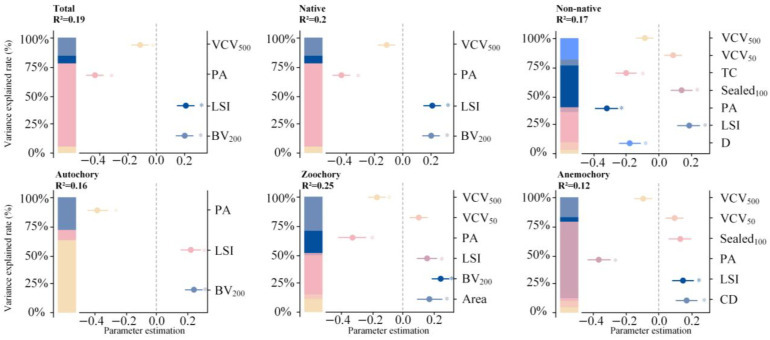
Variance decomposition of spontaneous plant richness.

Furthermore, both horizontal and vertical dimensions of urbanization jointly affected spontaneous plant richness. In the horizontal dimension, Sealed_100_ (the proportion of impervious surface within a 100 m buffer zone around the patches) was significantly positively correlated with the richness of non-native plants (*p* < 0.05; Figure 4; Table 1). For anemochorous plants, CD (Distance from patch to city center) positively correlates (*p* < 0.05) with the richness. In the vertical dimension, BV_200_ (total building volume within a 200 m buffer zone around the patches) was significantly positively correlated with the richness of total, native, autochorous, and zoochorous plants (*p* < 0.05). Additionally, VCV_500_ (building volume coefficient of variation around the patches within a 500 m radius) was significantly positively associated with the richness of total and zoochory (Figure 4; Table 1).

## 3. Discussion

### 3.1. Characterizing the Species Composition of Urban Spontaneous Plants

The floristic composition of spontaneous plants in Changchun reflects both the legacy of regional vegetation and the pressures of urbanization. The dominance of herbaceous life-forms suggests a high level of disturbance and frequent habitat turnover, which favors species with rapid life cycles and flexible reproductive strategies [34]. The prevalence of certain families, such as *Asteraceae*, *Poaceae*, and *Fabaceae*, highlights their adaptability to urban conditions and their ability to colonize fragmented or modified habitats [35]. These results were comparable to those of studies on spontaneous plants in other Chinese urban areas, such as Harbin, Zhengzhou, Wuhan, and Shanghai [26,36,37,38,39,40], and could be linked to the high reproductive and disturbance capabilities of these three families.

The dominance of herbaceous plants among the spontaneous flora in Changchun reflects the adaptive strategies these life-forms employ in response to urban environmental pressures. Perennial and annual/biennial herbs are particularly well-suited to disturbed, fragmented, and frequently changing habitats typical of urban landscapes. Their rapid growth, high reproductive output, and flexible dispersal mechanisms allow them to quickly colonize open or degraded sites, maintaining ecosystem functions in the face of ongoing disturbances [41].

A notable feature in Changchun is the relatively high proportion of native species (about 79%), which indicates a certain resilience of local flora in the face of urban pressures. This proportion is higher than reported in many European cities (e.g., Berlin, Vienna: 50–60% natives [42]), and North American cities such as New York (alien species exceed 30%; Aronson et al., 2014 [43]), but is similar to some Asian cities like Beijing [44]. Such differences may be shaped by regional landscape connectivity, historical introductions, and urban development intensity. Despite this, the presence of non-native and invasive species—especially those frequently encountered—remains a concern for biodiversity. Invasive plants, often from highly adaptable families like *Asteraceae*, can outcompete natives and alter ecosystem functions, reinforcing the need for ongoing monitoring and management [45,46]. Urbanization and greening projects may further facilitate their spread, while habitat degradation could limit native species reliant on specific conditions.

Dispersal mechanisms also play a significant role in shaping urban plant communities. The dominance of autochorous species points to restricted connectivity and limited animal-mediated seed dispersal, a trend consistent with findings from Nanjing and 16 cities in Yunnan province [9,47]. Urban infrastructure and simplified water systems reduce opportunities for long-distance dispersal, favoring plants capable of self-propagation [9].

### 3.2. Distribution Pattern and Causes of Urban Spontaneous Plant Diversity

The spatial patterns of spontaneous plant diversity across urban green space types reflect both ecological structure and the influence of human activities [48]. Residential green spaces supported the most diverse spontaneous plant communities, likely due to intentional planting, habitat complexity, and favorable soil and light conditions [49]. Such design and management practices not only enhance species richness but also promote a variety of community types. Similarly, square green spaces exhibited the highest diversity and evenness for spontaneous and native plants, possibly because their mixed vegetation structure—trees, shrubs, and grasses—offers multiple ecological niches [50]. Moderate anthropogenic management in these areas appears to maintain high diversity by balancing disturbance and preventing the dominance of a few species.

In contrast, brownfield and wasteland sites generally showed lower diversity indices, which may result from poor soil quality, contamination, and lack of vegetation structure [51]. Notably, the diversity of spontaneous non-native plants varied greatly in managed green spaces but remained low in protected and abandoned areas, highlighting the role of human intervention in shaping non-native species distributions [52]. At the habitat level, forest gaps provided optimal conditions—light, humidity, and temperature—for spontaneous plant richness, while scrub gaps supported high evenness and diversity among native plants, likely due to the coexistence of dominant and subordinate species. These findings underscore the importance of both ecological complexity and nuanced management in promoting urban plant diversity and highlight the need for targeted strategies to enhance native biodiversity while controlling non-native species [53].

### 3.3. Patch Characteristics and Edge Effects

Patch characteristics, such as shape complexity, size, and vegetation structure, are foundational drivers of urban plant diversity. A high landscape shape index (LSI) and greater perimeter–area ratio (PA) increase the number of edge habitats, facilitating species exchange and resource flow between patches [54]. These edges provide microhabitats with varying light, moisture, and temperature, supporting both native and non-native species with diverse ecological requirements. However, excessive fragmentation due to high PA can reduce core habitat quality, increase exposure to urban stressors, and promote the spread of invasive species [55].

Vegetation structure, particularly the coverage and layering of woody plants, also influences spontaneous plant richness. Dense canopy cover limits light availability at the ground level, suppressing the recruitment of light-demanding non-native species while favoring shade-tolerant natives [56]. Moreover, vertical stratification creates ecological niches for different functional groups, enhancing overall diversity. Active management of patch shape and vegetation layering—such as maintaining irregular patch edges and promoting multi-layered plantings—can optimize habitat heterogeneity and support urban biodiversity.

### 3.4. Joint Effect of Horizontal and Vertical Urbanization on Plant Richness

Urban structure, encompassing both horizontal and vertical dimensions, jointly shapes the composition and distribution of urban plant communities. The horizontal dimension is mainly reflected in the expansion of impervious surfaces and the spatial configuration of green spaces. Urban expansion often leads to habitat loss, isolation, and fragmentation, which can disrupt ecological connectivity and limit the dispersal of native species [57]. Our results confirm that increased imperviousness correlates with higher richness of non-native species, as disturbed, open areas favor the establishment and spread of exotics adapted to frequent disturbance and rapid colonization [58].

Maintaining horizontal connectivity through green corridors, stepping stones, and buffer zones is essential for supporting native species movement and ecological processes. Strategic use of spontaneous plants with diverse dispersal mechanisms—such as wind-dispersed pioneers in open spaces and animal-dispersed species in connected habitats—can enhance landscape resilience [59]. Urban planning should prioritize the integration of remnant natural habitats and the restoration of degraded sites to prevent biotic homogenization and loss of ecosystem services.

The vertical configuration of urban landscapes introduces a remarkable degree of environmental heterogeneity, which can profoundly shape plant community composition and diversity. Tall buildings and varying building volumes create a mosaic of microclimates by modifying sunlight exposure, temperature, humidity, and wind patterns at ground level. This spatial complexity provides a range of ecological niches, supporting not only shade-tolerant native species but also plants with diverse dispersal strategies. For instance, shaded areas beneath tall buildings may favor woodland perennials and understory species, while open, sunlit patches created by gaps in the built environment can support ruderal and pioneer species. The interplay of light and shade, coupled with variable thermal conditions, enhances the overall resilience and richness of urban plant assemblages [60].

Importantly, the variability in building volume across the urban matrix does more than alter abiotic conditions—it also influences the movement and behavior of animals that serve as pollinators and seed dispersers. Heterogeneous vertical structures provide diverse foraging, nesting, and sheltering opportunities for birds, insects, and small mammals. As a result, animal-dispersed (zoochorous) plant species are particularly well-positioned to thrive in areas with high architectural variability. The increased presence and activity of dispersal agents can facilitate the colonization and persistence of these plants, contributing to greater functional diversity within urban vegetation [61]. Moreover, the vertical complexity of buildings may extend growing seasons and buffer against extreme weather events, further supporting plant establishment and survival.

From a management perspective, these findings challenge the traditional notion that dense and variable urban structures are inherently detrimental to biodiversity. Instead, they underscore the potential for vertical architectural diversity to enhance habitat heterogeneity and ecological processes in cities. Urban planners and landscape architects can leverage this insight by integrating green infrastructure into vertical spaces—such as green roofs, living walls, and vegetated terraces—to amplify the ecological benefits of built environments. Such strategies not only support a broader array of plant and animal species but also contribute to ecosystem services, urban resilience, and human well-being.

### 3.5. Landscape Management Strategies for Urban Biodiversity Enhancement and Maintenance

The findings show that even the potentially harsh and fragmented environments of cities can harbor remarkable biodiversity. Spontaneous plants, with their natural ability to colonize and adapt to urban conditions, embody significant ecological, economic, and cultural values. Their resilience and adaptability make them ideal candidates for restoring and enhancing urban biodiversity, especially in areas where traditional landscaping may be challenging or resource-intensive [10,62]. Effective landscape management for urban biodiversity should build on these strengths by integrating both horizontal and vertical strategies. Horizontally, enhancing habitat connectivity through green corridors, stepping stones, and buffer zones enables the movement and persistence of native species. Protecting remnant habitats and restoring degraded sites with a diverse mix of native and spontaneous species can help maintain ecological processes and prevent biotic homogenization. Vertically, the use of green roofs, living walls, and vegetated terraces increases habitat heterogeneity and provides additional niches for plants and urban wildlife, further supporting ecosystem resilience.

Urban landscape management should prioritize the preservation of remnant near-natural habitats, such as wetlands and rivers, as metropolitan areas with these features tend to exhibit higher biodiversity indices [63]. It is critical to control urban development and maintain a particular proportion of natural habitats during the construction process. Artificially creating diverse near-natural habitats within various kinds of green spaces can serve as essential refuges for different flora and fauna. Our study indicates that biodiversity indices are higher in forest gaps and scrub gaps, emphasizing the importance of habitat heterogeneity. It is also important to manage spontaneous plants scientifically, change the prejudice against spontaneous plants, and take reasonable care and management measures. Scientific management of spontaneous plants is also crucial—appropriate interventions such as moderate pruning and the creation of spontaneous plant landscapes can improve plant diversity and ecosystem function, while overcoming prejudice against spontaneous vegetation [64].

Depending on the dispersal strategies of spontaneous plants, tailored management and application approaches are necessary [65]. Autochory, such as *Polygonum aviculare* and *Trigonotis peduncularis*, is suitable for small green spaces and building surroundings, but their spread should be controlled with physical barriers. Anemochory, such as *Taraxacum mongolicum*, can be used for rapid greening and wasteland restoration, with its propagation direction guided by wind patterns and landscape needs [66]. Zoochory, like *Duchesnea indica*, thrives in areas with abundant animal activity, and their distribution can be supported by creating food drop-off points and monitoring animal routes [67]. In landscaped areas with animal activity, animal routes can be guided by setting up food drop-off points, habitats, etc., and some animals that may be carrying exotic plant seeds can be monitored more closely. Hydrochory, such as *Phragmites australis*, is ideal for planting around urban water bodies, contributing to landscape beauty, water purification, and aquatic habitat provision. However, all these groups require careful monitoring to prevent excessive proliferation and maintain ecological balance.

A key aspect of sustaining urban biodiversity is the control of invasive plant species, which often take advantage of disturbed urban environments [68]. Our survey indicates that areas with frequent human disturbance tend to have a higher proportion of annual plants, many of which are invasive. This not only complicates landscape management but also threatens native plant populations, potentially leading to local extinctions. Therefore, introducing non-native plants must be preceded by rigorous risk assessment to evaluate their potential invasiveness. For instance, anemochory invasive plants like *Erigeron canadensis* have become dominant species in most patches in our study area, underscoring the need for caution when introducing such species. Management should focus on early detection and removal of invasives, promoting native and competitive spontaneous plants to fill ecological gaps, and raising public awareness about the impacts of invasive species [69]. Integrating these measures with broader landscape strategies ensures that urban ecosystems remain diverse, functional, and capable of providing essential services to both nature and people [70].

### 3.6. Limitations and Future Directions

This study reveals the patterns of spontaneous plant diversity distribution in urban environments; however, several limitations remain. First, as spontaneous plants include both summer and winter annuals, our single-season survey may introduce data bias. We plan to address this by conducting additional surveys in winter. Second, although we aimed to compare the effects of two-dimensional and three-dimensional urbanization on biodiversity, we did not account for soil physical and chemical properties, which may influence plant diversity. Future research will incorporate these factors into the analytical framework. Moreover, since urban species diversity can vary significantly across regions, more studies are needed that compare different cities horizontally and track long-term changes vertically. Such efforts will help us better understand the evolutionary patterns and drivers of spontaneous plant diversity in urban landscapes.

## 4. Materials and Methods

### 4.1. Study Area

**Figure 5:**
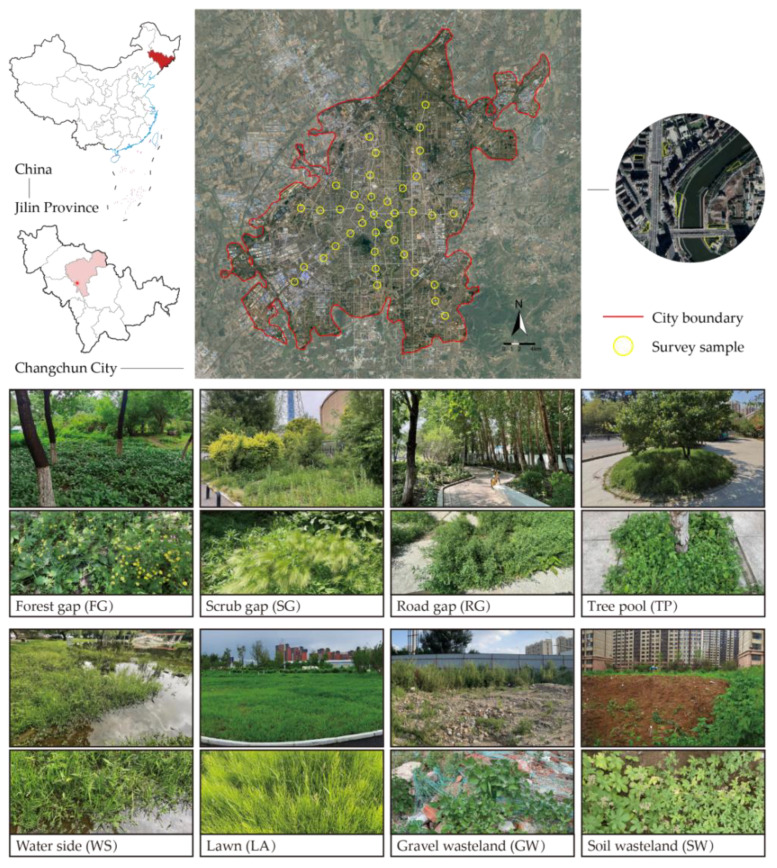
The green space types of patches and habitat types of plots.

Changchun City (124°18′ E–127°05′ E, 43°05′ N–45°15′ N, average elevation 215 m), the capital of Jilin Province, is situated in the middle region of the province and the hinterland of the Northeast China Plain (Figure 5). Changchun’s climate is typical of a temperate continental semi-humid monsoon. Changchun experiences four distinct seasons, with an average annual temperature of 6.5 °C, summer highs around 21.9 °C, and winter lows averaging −12.0 °C. The annual precipitation in Changchun typically ranges between 500 and 600 mm. By 2023, there will be over 8 million people living within a 676 km^2^ built-up area [30].

### 4.2. Field Survey

The urban boundary was delineated using 2020 ESA World Cover Sentinel-2 data, identifying areas with impervious surfaces covering ≥50% through a 1 km × 1 km moving window analysis in Fragstats 4.2 [71]. Eight radial transects were set up from Changchun’s city center, with circular sample sites (400 m radius) placed every 2 km, totaling 38 sites. In each survey site, 6–7 accessible urban green space patches were randomly selected for detailed plant community surveys. Moreover, the selected patches will cover as many different types of green spaces as possible to ensure that no data are omitted. These surveys documented the woody layer coverage and the type of green space in each patch. A total of 245 urban green space patches were surveyed from June to August in 2023 and 2024. In each patch, at least two herbaceous plots with a 1 m × 1 m size were established for typical community sampling, recording the plant species present, their maximum height and cover, the overall height and cover of the community, and the habitat type (Figure 5; Table 2). In total, 1147 herbaceous plots were surveyed.

The spontaneous plants in this study were defined as regenerated tree and shrub seedlings, as well as spontaneous herbaceous plants. Spontaneous herbaceous plants were naturally occurring herbaceous plants which have traditionally been described as “weeds”, “ruderal plants”, or “escaped plants” and could be easily distinguished from the cultivated planted species. And the seedlings of woody plants can be clearly distinguished from cultivated plants in all urban manmade green patches.

### 4.3. Data Analysis

#### 4.3.1. Features of Spontaneous Plant Classification

Plant identification of all patches was performed according to the Chinese Plant Science Data (https://www.iplant.cn/, accessed on 15 May 2023). The spontaneous plants were classified into native and non-native plants based on the Chinese List of Invasive and Naturalized Plants (2023 Edition) [72] and a dataset on the catalog of alien plants in China [73]. The seed dispersal modes of species are typically classified into four categories: anemochory, autochory, hydrochory, or zoochory [74]. The seed dispersal modes of species are identified through seed information databases (https://ser-sid.org/, accessed on 1 August 2023).

#### 4.3.2. Species Diversity Analysis

The Richness index, Shannon-Wiener diversity index, Pielou evenness index, and Simpson’s index were used to represent the diversity status of each plot. The diversity indexes were computed by using the “vegan” package in R. Using the Kruskal–Wallis test with significance levels adjusted by the Bonferroni method, differences in diversity between habitat types and under various urbanization gradients were compared. The test procedure was calculated using the R 4.3.1 package rstatix.

#### 4.3.3. Selection of Drivers for Species Richness

The independent factors affecting plant diversity in this study were classified into two categories: patch characteristic factors and urbanization characteristic factors. The patch characteristic factors included patch area (Area), patch’s perimeter–area ratio (PA), landscape shape index (LSI), the woody layer cover of the patch (TC), and disturbance intensity (D). Urbanization characterization factors included the distance from patch to city center (CD), the proportion of impervious surface within a 100 m buffer zone around the patches (Sealed_100_), total building volume within a 200 m buffer zone around the patches (BV_200_), and building volume coefficient of variation around the patches within 50- and 500 m radii (VCV_50_, VCV_500_). A total of 10 factors were selected (Table 3).

#### 4.3.4. Statistical Data Analysis

First, the “corplot” function from the “corplot” package was used to perform pairwise Spearman correlation tests on the 10 driving factors. To avoid high collinearity, driving factors with correlation coefficients greater than 0.7 were screened, and we retained the variable with greater theoretical relevance or explanatory power. Model fitting was conducted using the “lm” function from the “stats” package, and the “dredge” function from the “MuMIn” package was employed to perform full subset selection on the full model, with the best model selected based on the AICc (corrected Akaike’s Information Criterion) criterion. The individual explanatory power of each driving factor was assessed using variance partitioning via the “rdacca.hp” package to obtain the relative contribution of each variable. All the analyses were performed using R software 4.3.1 [75].

## 5. Conclusions

This study systematically elucidates the spatial patterns and driving factors of spontaneous plant diversity in a cold climate megacity under multidimensional urbanization. By integrating both horizontal (e.g., land use, impervious surface ratio, distance to city center) and vertical (e.g., building volume, building height) urbanization metrics, we found that spontaneous plants are capable of thriving even in highly urbanized and fragmented environments, with native herbs as the dominant life-form. Vertical urbanization, particularly building volume, was positively associated with overall species richness, while horizontal factors more strongly influenced non-native and wind-dispersed species. Significant differences in plant diversity indices were observed among various habitat types, emphasizing the importance of habitat heterogeneity and the preservation of remnant natural spaces. The study underscores the need for integrating multidimensional urbanization perspectives into urban planning and green space management to mitigate biodiversity loss and enhance ecological functions.

Despite certain limitations, such as single-season sampling and the lack of soil property analysis, this research offers new insights into the complex interplay between urbanization and biodiversity. Future studies should address these gaps through multi-seasonal surveys, incorporation of soil factors, and broader regional comparisons to fully understand the evolutionary dynamics of spontaneous plant diversity. Overall, our work provides a theoretical foundation for sustainable urban planning and the integration of biodiversity conservation into the ongoing transformation of urban landscapes.

## Figures and Tables

**Table 1:** Predicted results of linear regression models for species richness at the urban scale. For the abbreviations, please see Figure 4. Bold font means significance (*p* < 0.05).

	Driving Factors	Estimate	t Value	SE	*R* ^2^	Importance
Total: *R*^2^ = 0.20 adjusted *R*^2^ = 0.19	PA	**−0.4378**	−6.9360	0.0631	0.1385	0.4593
	LSI	**0.2038**	3.2099	0.0635	0.0123	0.2138
	BV_200_	**0.1981**	3.4122	0.0580	0.0296	0.2078
	VCV_500_	**−0.1134**	−1.9693	0.0576	0.0095	0.1190
Native: *R*^2^ = 0.21 adjusted *R*^2^ = 0.20	PA	**−0.4509**	−7.1191	0.0626	0.1492	0.4665
	LSI	**0.1956**	3.1055	0.0630	0.0109	0.2024
	BV_200_	**0.2086**	3.6213	0.0576	0.0336	0.2159
	VCV_500_	−0.1113	−1.9477	0.0572	0.0091	0.1152
Non-native plants: *R*^2^ = 0.19 adjusted *R*^2^ = 0.17	PA	**−0.3241**	−4.7862	0.0677	0.0601	0.2653
	LSI	**0.1772**	2.6295	0.0674	0.0091	0.1451
	TC	**−0.2065**	−3.4588	0.0597	0.0434	0.1691
	D	**−0.1798**	−3.0264	0.0594	0.0302	0.1472
	Sealed_100_	**0.1352**	2.0833	0.0649	0.0080	0.1107
	VCV_50_	0.1109	1.8208	0.0609	0.0112	0.0908
	VCV_500_	−0.0874	−1.4866	0.0588	0.0041	0.0715
Autochory: *R*^2^ = 0.17 adjusted *R*^2^ = 0.16	PA	**−0.3854**	−5.9979	0.0642	0.1011	0.4596
	LSI	**0.2158**	3.3393	0.0646	0.0147	0.2574
	BV_200_	**0.2372**	4.0138	0.0591	0.0449	0.2829
Zoochory: *R*^2^ = 0.27 adjusted *R*^2^ = 0.25	Area	**0.1649**	1.9795	0.0833	0.0759	0.1461
	PA	**−0.3223**	−3.5303	0.0913	0.0870	0.2855
	LSI	**0.1468**	2.2568	0.0650	0.0058	0.1300
	BV_200_	**0.2342**	4.1176	0.0568	0.0495	0.2075
	VCV_50_	0.0952	1.6679	0.0571	0.0093	0.0844
	VCV_500_	**−0.1651**	−2.9799	0.0554	0.0248	0.1463
Anemochory: *R*^2^ = 0.14 adjusted *R*^2^ = 0.12	PA	**−0.3592**	−5.1587	0.0696	0.0775	0.3630
	LSI	**0.1487**	2.1789	0.0682	0.0038	0.1502
	CD	**0.1674**	2.7569	0.0607	0.0203	0.1692
	Sealed_100_	0.1304	1.9494	0.0669	0.0028	0.1317
	VCV_50_	0.0918	1.4660	0.0626	0.0065	0.0928
	VCV_500_	−0.0918	−1.5241	0.0602	0.0046	0.0928

**Table 2:** The green space types of patches and habitat types of plots.

Types of Green Spaces	Number of Patches	Habitat Types	Number of Plots
Park green space (P-G)	40	Forest gap (FG)	389
Road green space (R-G)	52	Scrub gap (SG)	312
Affiliated green space (I-G)	30	Road gap (RG)	221
Residential green space (RE-G)	102	Tree pool (TP)	13
Square green space (PL-G)	11	Water side (WS)	22
Brownfield site (B-F)	9	Lawn (LA)	146
Protected green space (PR-G)	1	Gravel wasteland (GW)	21
		Soil wasteland (SW)	23

**Table 3:** Characterization factors.

	Characterization Factors	Formula
Patch characteristic factors	Patch area (Area)	
	Patch’s perimeter–area ratio (PA)	*PA = Perimeter/Area*
	Landscape shape index (LSI)	$LSI=Perimeter/2\sqrt{\pi \times A}$
	The woody layer cover of the patch (TC)	TC = Area of woody plants/Area of patch, proportion of plants in each patch
	Disturbance intensity (D)	
Urbanization characterization factors	Distance from patch to city center (CD)	
	The proportion of impervious surface within a 100-m buffer zone around the patches (Sealed_100_)	Sealed._patch_ = The area sealed/Total area of site, proportion of sealed surface in each patch
	Total building volume within a 200-m buffer zone around the patches (BV_200_)	BV._patch_ = The product of building height and building area within the patch buffer
	Building volume coefficient of variation around the patches within different radii (VCV_50_, VCV_500_)	VCV._patch_ = Standard deviation of building volume/Average value of building volume

## Data Availability

The data presented in this study are available from the corresponding author upon reasonable request.

## Supplementary Materials

The following supporting information can be downloaded at https://doi.org/10.5281/zenodo.13907273.

## References

[1] Groffman P.M., Cavender-Bares J., Bettez N.D., Grove J.M., Hall S.J., Heffernan J.B., Hobbie S.E., Larson K.L., Morse J.L., Neill C. (2014). Ecological homogenization of urban USA. Front. Ecol. Environ..

[2] Zhao S., Da L., Tang Z., Fang H., Song K., Fang J. (2006). Ecological consequences of rapid urban expansion: Shanghai, China. Front. Ecol. Environ..

[3] Norton B.A., Evans K.L., Warren P.H. (2016). Urban Biodiversity and Landscape Ecology: Patterns, Processes and Planning. Curr. Landsc. Ecol. Rep..

[4] Yu D., Wang D., Li W., Liu S., Zhu Y., Wu W., Zhou Y. (2018). Decreased Landscape Ecological Security of Peri-Urban Cultivated Land Following Rapid Urbanization: An Impediment to Sustainable Agriculture. Sustainability.

[5] Bai L., Xiu C., Feng X., Liu D. (2019). Influence of urbanization on regional habitat quality:a case study of Changchun City. Habitat Int..

[6] Marselle M.R., Lindley S.J., Cook P.A., Bonn A. (2021). Biodiversity and Health in the Urban Environment. Curr. Environ. Health Rep..

[7] Wang D., Xu P.-Y., An B.-W., Guo Q.-P. (2024). Urban green infrastructure: Bridging biodiversity conservation and sustainable urban development through adaptive management approach. Front. Ecol. Evol..

[8] Gao Z., Pan Y., Bodegom P.M.V., Cieraad E., Xing D., Yang Y., Xia T., Luo X., Song K., Da L. (2023). Beta diversity of urban spontaneous plants and its drivers in 9 major cities of Yunnan province, China. Landsc. Urban Plan..

[9] Gao Z., Pan Y., Song K., Yang Y., Zhuge M., Wu T., Xia T., Hu Y., Da L., Cieraad E. (2024). Response and sensitivity of urban plants with different seed dispersal modes. Nat. Cities.

[10] Gao Z.W., Song K., Pan Y.J., Malkinson D., Zhang X.J., Jia B., Xia T.Y., Guo X.Y., Liang H., Huang S.S. (2021). Drivers of spontaneous plant richness patterns in urban green space within a biodiversity hotspot. Urban For. Urban Green..

[11] Prach K., Bartha S., Joyce C.B., Pyšek P., Diggelen R.v., Wiegleb G. (2001). The Role of Spontaneous Vegetation Succession in Ecosystem Restoration: A Perspective. Appl. Veg. Sci..

[12] Chen C.D. (2020). Forgotten urban habitats: Analysis of spontaneous vegetation on the urban walls of Chongqing City. Acta Ecol. Sin..

[13] Huang L., Qian S.H., Li T., Jim C.Y., Jin C., Zhao L., Lin D.M., Shang K.K., Yang Y.C. (2019). Masonry walls as sieve of urban plant assemblages and refugia of native species in Chongqing, China. Landsc. Urban Plan..

[14] Li D., Wen L., Dong R.R., Hu Y.D. (2024). Diversity and distribution characteristics of spontaneous plants in urban industrial wasteland in Harbin. Landsc. Archit..

[15] Liang X.Y., You J.X., Zhu S., Hu Y.D. (2023). Spontaneous vegetation species diversity and distribution in heterogeneous habitats of university campus green spaces of Harbin. Chin. Landsc. Archit..

[16] Kowarik I. (2011). Novel urban ecosystems, biodiversity, and conservation. Environ. Pollut..

[17] Oberndorfer E., Lundholm J., Bass B., Coffman R.R., Doshi H., Dunnett N., Gaffin S., Koehler M., Liu K.K.Y., Rowe B. (2007). Green roofs as urban ecosystems: Ecological structures, functions, and services. Bioscience.

[18] Zhang L.L., Hao P.Y., Dong L., Wang Y.L. (2024). Optimization Strategy for Maintenance Management of Herbaceous Layer in Urban Parks Based on Spontaneous Plants: A Case Study of Xicheng District, Beijing. Landsc. Archit..

[19] Aronson M.F.J., La Sorte F.A., Nilon C.H., Katti M., Goddard M.A., Lepczyk C.A., Warren P.S., Williams N.S.G., Cilliers S., Clarkson B. (2014). A global analysis of the impacts of urbanization on bird and plant diversity reveals key anthropogenic drivers. Proc. R. Soc. B-Biol. Sci..

[20] Chen X.S. (2014). Distribution Patterns of Ruderal Communities and its Responses to Habitat Heterogeneity in Urban Area of Harbin. Ph.D. Thesis.

[21] Rega-Brodsky C.C., Aronson M.F.J., Piana M.R., Carpenter E.-S., Hahs A.K., Herrera-Montes A., Knapp S., Kotze D.J., Lepczyk C.A., Moretti M. (2022). Urban biodiversity: State of the science and future directions. Urban Ecosyst..

[22] Liu R., Yan X., Lin X., Sun Y., Zhang T., Xiao J. (2023). Urban spontaneous plant richness in response to the 2D/3D building and green space patterns in a highly urbanized area. Ecol. Indic..

[23] Malkinson D., Kopel D., Wittenberg L. (2018). From rural-urban gradients to patch—Matrix frameworks: Plant diversity patterns in urban landscapes. Landsc. Urban Plan..

[24] Kondratyeva A., Knapp S., Durka W., Kühn I., Vallet J., Machon N., Martin G., Motard E., Grandcolas P., Pavoine S. (2020). Urbanization Effects on Biodiversity Revealed by a Two-Scale Analysis of Species Functional Uniqueness vs. Redundancy. Front. Ecol. Evol..

[25] Xu Q., Zheng X., Zhang C. (2018). Quantitative Analysis of the Determinants Influencing Urban Expansion: A Case Study in Beijing, China. Sustainability.

[26] Jia B. (2021). Plant Diversity and Community Types of Urban Green Space on Urban-Rural Gradient in Harbin. Master’s Thesis.

[27] Su M., Jie P., Li P., Yang F., Huang Z., Shi X. (2024). A review on the mechanisms behind thermal effect of building vertical greenery systems (VGS): Methodology, performance and impact factors. Energy Build..

[28] Chang Y., Wang Z., Zhang D., Fu Y., Zhai C., Wang T., Yang Y., Wu J. (2022). Analysis of Urban Woody Plant Diversity among Different Administrative Districts and the Enhancement Strategy in Changchun City, China. Sustainability.

[29] Zhang D., Zheng H., He X., Ren Z., Zhai C., Yu X., Mao Z., Wang P. (2015). Effects of forest type and urbanization on species composition and diversity of urban forest in Changchun, Northeast China. Urban Ecosyst..

[30] Dong L.D. (2024). Changchun Statistical Yearbook.

[31] Chang S., Su K., Jiang X., You Y., Li C., Wang L. (2023). Impacts and Predictions of Urban Expansion on Habitat Connectivity Networks: A Multi-Scenario Simulation Approach. Forests.

[32] Nowak D.J. (2010). Urban Biodiversity and Climate Change. Urban Biodiversity and Design.

[33] Chen C., Mao L., Qiu Y., Cui J., Wang Y. (2020). Walls offer potential to improve urban biodiversity. Sci. Rep..

[34] Prochazka L.S., Alcantara S., Rando J.G., Vasconcelos T., Pizzardo R.C., Nogueira A. (2024). Resource availability and disturbance frequency shape evolution of plant life forms in Neotropical habitats. New Phytol..

[35] Benvenuti S. (2004). Weed dynamics in the Mediterranean urban ecosystem: Ecology, biodiversity and management. Weed Res..

[36] Chen X.S., Wang W.B., Liang H., Liu X.L., Da L.J. (2014). Dynamics of ruderal species diversity under the rapid urbanization over the past half century in Harbin, Northeast China. Urban Ecosyst..

[37] Dubois J., Cheptou P.-O. (2017). Effects of fragmentation on plant adaptation to urban environments. Philos. Trans. R. Soc. London. Ser. B Biol. Sci..

[38] Jiang H.Q. (2023). Distribution of Spontaneous Plants in the Central City of Wuhan and Its Influencing Factor. Master’s Thesis.

[39] Tian Z.H. (2011). Study on Distribution Patterns of Weed Communities of Terrestrial Ecosystem and Forming Reason in Urban and Rural of Shanghai. Ph.D. Thesis.

[40] You Q. (2023). Composition and Community Characteristics of Spontaneous Herbaceous Plants in the Main Urban Area of Zhengzhou. Master’s Thesis.

[41] Herben T., Klimešová J., Chytrý M. (2018). Effects of disturbance frequency and severity on plant traits: An assessment across a temperate flora. Funct. Ecol..

[42] Kühn I., Klotz S. (2006). Urbanization and homogenization—Comparing the floras of urban and rural areas in Germany. Biol. Conserv..

[43] Aronson M.F.J., Handel S.N., La Puma I.P., Clemants S.E. (2014). Urbanization promotes non-native woody species and diverse plant assemblages in the New York metropolitan region. Urban Ecosyst..

[44] Wang K. (2014). Research on the Spontaneous Herbaceous Plants in Beijing Urban Area. Master’s Thesis.

[45] Pyšek P., Richardson D.M. (2010). Invasive Species, Environmental Change and Management, and Health. Annu. Rev. Environ. Resour..

[46] Gaertner M., Novoa A., Fried J., Richardson D.M. (2017). Managing invasive species in cities: A decision support framework applied to Cape Town. Biol. Invasions.

[47] Zhang M., Li Q., Li Z. (2020). Seed dispersal modes and landscape application strategies of autogenesis herbs in the parks of Nanjing City. Chin. Landsc. Archit..

[48] Hope D., Gries C., Casagrande D., Redman C.L., Grimm N.B., Martin C. (2006). Drivers of Spatial Variation in Plant Diversity Across the Central Arizona-Phoenix Ecosystem. Soc. Nat. Resour..

[49] Ran C., Pan J., Lin Y., Li T., Huang Y., Huang J., Fan S., Fang W., Zhao S., Liu Y. (2024). Utilizing spontaneous plants for sustainable development in residential green spaces: Insights from environmental drivers and niche analysis in Fuzhou City, China. J. Environ. Manag..

[50] Threlfall C.G., Ossola A., Hahs A.K., Williams N.S.G., Wilson L., Livesley S.J. (2016). Variation in Vegetation Structure and Composition across Urban Green Space Types. Front. Ecol. Evol..

[51] Singh D.K., Singh A., Gacem A., Kashyap S., Yadav V.K., Yadav K.K., Hussein H.S., Shukla N.K., Alsuhaibani A.M., Abdellattif M.H. (2023). Multiple Site Dissimilarities of Herbaceous Species Due to Coal Fly Ash Dumping Based Soil Heavy Metal Toxication. Toxics.

[52] Tredici P.D. (2010). Spontaneous Urban Vegetation: Reflections of Change in a Globalized World. Nat. Cult..

[53] Xie C., Chen S., Liu D., Jim C.Y. (2024). Unveiling the complex networks of urban tree diversity research: A global perspective. Ecol. Evol..

[54] Heegaard E., Økland R.H., Bratli H., Dramstad W.E., Engan G., Pedersen O., Solstad H. (2007). Regularity of species richness relationships to patch size and shape. Ecography.

[55] Minor E.S., Tessel S.M., Engelhardt K.A.M., Lookingbill T.R. (2009). The role of landscape connectivity in assembling exotic plant communities: A network analysis. Ecology.

[56] Connolly B.M., Powers J., Mack R.N. (2017). Biotic constraints on the establishment and performance of native, naturalized, and invasive plants in Pacific Northwest (USA) steppe and forest. NeoBiota.

[57] Fischer J., Lindenmayer D.B. (2007). Landscape modification and habitat fragmentation: A synthesis. Glob. Ecol. Biogeogr..

[58] Jesson L., Kelly D., Sparrow A. (2000). The importance of dispersal, disturbance, and competition for exotic plant invasions in Arthur’s Pass National Park, New Zealand. N. Z. J. Bot..

[59] Uroy L., Mony C., Ernoult A. (2019). Additive effects of connectivity provided by different habitat types drive plant assembly. Sci. Rep..

[60] Ren Y., Guo M., Yin F., Zhang M.-J., Wei J. (2022). Tree Cover Improved the Species Diversity of Understory Spontaneous Herbs in a Small City. Forests.

[61] Borowy D., Swan C.M. (2022). The effects of local filtering processes on the structure and functioning of native plant communities in experimental urban habitats. Ecol. Evol..

[62] Zhang M.Y., Li X.L., Fan S.X., Li K., Xing X.Y., Xu Y.D., Hao P.Y., Dong L. (2024). Response of spontaneous plant communities to microhabitats in a riparian corridor in Beijing, China. Sci. Rep..

[63] Hu Y.D., S K.K. (2023). Create Habitats to Boost Biodiversity in Urban Greening. Science.

[64] Kühn N. (2006). Intentions for the Unintentional. J. Landsc. Archit..

[65] Huang J.L., Qian S.H., Fortin M.J. (2025). Spatiotemporal land use dynamics filter life history strategies to shape urban spontaneous plant assemblages. Ecol. Appl..

[66] Zhu C., Lu J., Ma X., Wang D. (2024). Constructing a Multivariate Linear Model to Investigate the Wind Propagation Dynamics of Dandelion with Analytic Hierarchy Process. Acad. J. Sci. Technol..

[67] Gelmi-Candusso T.A., Hämäläinen A.M. (2019). Seeds and the City: The Interdependence of Zoochory and Ecosystem Dynamics in Urban Environments. Front. Ecol. Evol..

[68] Francis R.A., Chadwick M.A. (2015). Urban invasions: Non-native and invasive species in cities. Geography.

[69] Hobbs R.J., Humphries S.E. (1995). An Integrated Approach to the Ecology and Management of Plant Invasions. Conserv. Biol..

[70] Haase D., Weith T., Barkmann T., Gaasch N., Rogga S., Strauß C., Zscheischler J. (2021). Integrating Ecosystem Services, Green Infrastructure and Nature-Based Solutions—New Perspectives in Sustainable Urban Land Management. Sustainable Land Management in a European Context: A Co-Design Approach.

[71] Mcgarigal K. (2015). FRAGSTATS Help.

[72] Qin F., Xue T.-T., Liang Y.-F., Zhang W.-D., Liu Q., Chen T.-X., Bussmann R.W., Han B.-C., Yu S.-X. (2024). Present status, future trends, and control strategies of invasive alien plants in China affected by human activities and climate change. Ecography.

[73] Lin Q.W., Xiao C., Ma J.S. (2022). A dataset on catalogue of alien plants in China. Biodivers. Sci..

[74] E.D (1983). Principles of dispersal in higher plants. Biol. Conserv..

[75] Sohil F., Sohali M.U., Shabbir J. (2021). An introduction to statistical learning with applications in R. Stat. Theory Relat. Fields.

